# The association of lipid metabolism relative gene polymorphisms and ischemic stroke in Han and Uighur population of Xinjiang

**DOI:** 10.1186/s12944-017-0491-9

**Published:** 2017-06-17

**Authors:** Yun-hua Yue, Ling-yun Liu, Liang Hu, You-mei Li, Jie-ping Mao, Xiao-ying Yang, Na-mu Dila

**Affiliations:** 1 Department of Neurology, Yangpu Hospital Tongji University School of Medicine, No. 450 Tengyue Road, Shanghai, 200090 China; 2 Department of Neurology, Friendship Hospital to Urumqi, Urumqi, 830049 China

**Keywords:** Apolipoprotein, Low density lipoprotein receptor, Ischemic stroke, Single nucleotide polymorphism

## Abstract

**Background:**

The present study is aimed to evaluate difference of lipid metabolism related gene single nucleotide polymorphisms (SNPs) with ischemic stroke (IS) in Han and Uighur population of Xinjiang, China.

**Methods:**

Four hundred eight patients with ischemic stroke and 347 unrelated healthy individuals of age and sex matched were genotyped for Apolipoprotein A5 (ApoA5), lipoprotein lipase (LPL), Cholesteryl ester transfer protein (CETP) and low-density lipoprotein receptor (LDL-R) genes. Their mutation difference was analyzed by SNaP shot techniques. GeneMapper4.1 SPSS20.0 software was used for data management and analysis. Using a single locus analysis, the distribution difference of genotype loci in ischemic stroke cases and controls were detected to assess the genetic risk factors of ischemic stroke.

**Results:**

Significance differences of genotype distribution in ischemic stroke cases and controls were observed in LDLR rs688 in Han and Uighur population in recessive model from analysis of single gene locus. It also was found that dramatic difference of triglyceride (TG) of LPL rs328 and systolic blood pressure in CETP rs708277 of total population. In binary logistic regression analysis of total studied population, ischemic stroke was observed significantly associated with LDLR rs688 both addictive model (TT/CC, adjusted OR = 1.47, 95% CI = 1.04–2.07) and recessive model (TT/CT + CC, adjusted Odds ratio (OR) = 2.66, 95% Confidence Interval (CI) = 1.37–5.14). In Han population, ischemic stroke was observed significantly associated with rs688 both in addictive model (TT/CC, adjusted OR = 3.27, 95% CI = 1.06–10.05). In Uighur population, no significant association was found between gene polymorphisms and the risk of ischemic stroke. Combined analysis of multiple gene and loci, interaction effects of LDLR rs688 C/T, ApoA5 rs662799 A/G and CETP rs708272 C/T denoted a significant influence on IS susceptibility.

**Conclusion:**

Single nucleotide polymorphisms of lipid metabolism relative gene were significantly associated with the morbidity of ischemic stroke in Han population. The interaction effects of rs688 C/T with ApoA5 rs662799 A/G and CETP rs708272 C/T promoted the occurrence of IS.

## Background

Stroke is one of the leading causes of death over the all world and cause major health problem [1]. However, it is the first cause of morality in China [2]. Ischemic stroke (IS) frequently caused dysfunction of the brain and comprises 80% of cases [3]. The reasons for the patho-physiological cause of ischemic stroke are unclear. The concept was widely accepted that stroke is caused by combination of genetic and environment factors. Among genetic factors, it well known that a few specific gene variants of lipid metabolism are modifiable risk factors for the occurrence of IS [1, 4]. The extensive study indicated that atherosclerotic plaques form is the major cause of IS [5, 6], which the occurrence of atherosclerotic plaques are strongly associated with abnormal lipid metabolism [7, 8]. So far, it was known that Apolipoprotein A5 (ApoA5), lipoprotein lipase (LPL), Cholesteryl ester transfer protein (CETP) and low-density lipoprotein receptor (LDL-R) gene mutations had increased the concentration of lipids [9–11].

The association of human candidate genes with IS patients may be the confounding effects of ethnic, environment factor and individual life style [12]. Xinjiang region of China lies in the north-west part of the mainland and the weather is dry and cold. There is a high altitude in this area and relative lack of oxygen. Furthermore, the Uighur population has different risk factors of ischemic stroke than Han population because of the ethnicity, genetic background and lifestyle. The most population in Xinjiang had more caloric food and alcoholic intake [13, 14]. Hu et al. reported that the risk of dyslipidemia in CETP rs708272 CC of Uighur residents was much higher that rs708272 CT or TT genotype [15]. Quan et al. found that the patients with impaired fasting glucose (IFG) in Uighur population had more risk for dyslipidemia than Han population with IFG [16]. Li et al. showed that the level of hypercholesterolemia in Kazakh population of Xinjiang was significant higher than it in Uygur and Han [17]. These data indicated that different genetic background had more effect on their serum lipids level. Therefore, we determined to investigate ApoA5, LPL, CETP and LDLR gene single nucleotide polymorphisms (SNPs) in Han and Uighur population of Xinjiang region because of their different ethnicity, genetic, environment factors background and life style. These results will provided a unique link between the relationships the lipid metabolism related gene SNP with the occurrence of IS.

## Methods

### Study subjects

Patients with ischemic stroke of 250 Han and 158 Uighur population and were enrolled from Department of Neurology of the Youyi Hospital of Wulumuqi from July 2010 to July 2012. Three hundred forty-seven age and sex matched unrelated healthy individuals also were included in this study as a control The inclusion and exclusion criteria for ischemic stroke were determined as our previous described [18]. The hypertension and Diabetes mellitus (DM) diagnosis criteria were described as our and the other lab [18, 19]. Briefly, blood pressure (BP) was measured at routine method. The second BP with the fifth-phase diastolic pressure was used for analysis. Diabetes mellitus (DM) was classified as fasting plasma glucose (FPG) ≥7.0 mmol/L and/or 2 h plasma glucose (PG) ≥11.1 mmol/L or already diagnosed as diabetes patients. To assess the relationship of hypertension and DM with dislipidemia, the lipid level in IS patients with hypertension and DM was evaluated. This study protocol was approved by the Youyi hospital ethics committee and all participants were given signed informed consent.

### DNA extraction

5 ml of peripheral whole blood was collected in an EDTA coated vacutainerand blood samples were stored at 4 °C before DNA extraction. The genomic DNA was isolated using Qiagen DNA mini isolation kit. All procedure was performed according to kit description. DNA concentration and purity were determined by Nano Drop 2000 machine (Thermo Scientific, USA).

### Selection of SNP candidate genes

To identify the most likely relative lipoprotein SNP candidate genes, we accessed to http://www.ncbi.nlm.nih.gov/SNP database and selected APOA5, LPL, CETP and LDLR genes asour candidate genes association with acute IS.

### Genotyping of the ApoA5, LPL, CETP and LDLR gene SNPs

DNA sample were analyzed by SNaPshot SNP typing machine (SnaPshot multiplex, ABI, USA). Detail PCR and single-base extension primer sequences were recorded as our previous study [18].

### Statistical analysis

The statistical analysis was performed using SPSS 20.0(IBM, USA). Clinical parameter such as serum TC, TGL, HDL-C, LDL-C, ApoA1, ApoB, Lpa glucose, and serumwere measured with automatic machine in IS patients and controls. The allelic frequencies were calculated by Hardy-weinberg equipment method. Multivariate logistic regression analyses were performed for genotype, allele frequencies and lipid profile factor associated with ischemic stroke. Through counting DNA sequencing data, the genotype and allele frequencies can be estimated. The distinction between studied groups were analyzed by Pearson’s X^2^ test. Then, logistic regression analysis was measured the strength.

## Results

### Clinical character of subjects

The basic clinical characteristics of the 408 IS patients and 347 control were shown in Table 1. There were no significant difference in age, sex and body mass index (BMI), However, it was greatly differences in hypertension, diabetes mellitus (DM); Total Cholesterol (TC); Triglycerides (TG); low Density Lipoprotein-C (LDL-C); High Density Lipoprotein-C (HDL-C); Apolipoprotein–A1 (ApoA1); Apolipoprotein B (ApoB); lipoprotein a (Lpa) (*P* < 0.001). Therefore, these clinical parameters provide an evidence that IS patients often have abnormal serum lipoprotein level. It also is closed association with hypertension and DM history.

**Table 1:** Candidate genes and SNP information

Gene	IS group (*n* = 408)	Control group (*n* = 347)	*P*
Age (year-old)	61.9 ± 11.8	61.8 ± 11.7	0.916
Sex (Male/Female, *N*)	242/166	201/146	0.943
BMI (kg/m2)	24.2 ± 3.3	23.9 ± 4.1	0.542
Hypertension (*N*, %)	281(68.9)	129(37.2)	<0.001
DM (*N*, %)	129 (31.6)	46(13.3)	<0.001
TC (mmol/L)	4.85 ± 1.08	4.47 ± 1.01	<0.001
TG (mmol/L)	1.71 ± 0.65	1.51 ± 0.66	<0.001
LDL-C (mmol/L)	3.28 ± 0.61	2.46 ± 0.63	<0.001
HDL-C (mmol/L)	1.06 ± 0.25	1.27 ± 0.26	<0.001
ApoA1 (g/L)	0.83 ± 0.22	1.03 ± 0.31	<0.001
ApoB (g/L)	0.81 ± 0.22	0.95 ± 0.35	<0.001
Lpa (mg/L)	173.57 ± 53.17	123.81 ± 62.43	<0.001

*SNP* single nucleotide polymorphism, *BMI* Body Mass Index, *IS* ischemic stroke, *DM* Diabetes mellitus, *TC* Total Cholesterol, *TG* Triglycerides, *LDL-C* low Density Lipoprotein-C, *HDL-C* High Density Lipoprotein-C, *ApoA1* Apolipoprotein–A1, *ApoB* Apolipoprotein B, *Lpa* lipoprotein a

### Lipidsmetabolism relative genes SNP information

After we accessed to http://www.ncbi.nlm.nih.gov/SNP database a, it was found that possible APOA5, LPL, CETP and LDLR genes candidate SNP site were shown in Table 2. These mutations were verified by PCR and DNA product sequencing. These data confirmed that these candidate genes were indeed mutated in IS patients.

**Table 2:** Candidate Genes and SNP information

Gene	Locus	Function	Sites	HF	LF	Reference
APOA5	rs662799	5′-flanking	116,663,707	A	G	0.267(HCB)
LPL	rs320	Intron	19,819,077	T	G	NA(HCB)
	rs328	Ser474Ter	19,819,724	C	G	0.089(HCB)
CETP	rs708277	Intron	56,996,288	C	T	NA(HCB)
LDLR	rs688	Asn591Asn	11,227,602	C	T	0.174(HCB)

*SNP* single nucleotide polymorphism, *ApoA5* Apolipoprotein–A5, *LPL* lipoprotein lipase, *CETP* Cholesteryl ester transfer protein, *LDLR* Low density lipoprotein receptor, *HF* high frequency, *LF* low frequency, *NA* no answer, *HCB* Han, Chinese and Beijing

### The distribution difference of ApoA5 rs662799, LPL rs320, rs328, CETP rs708277, LDLR rs688 mutation with IS in Han and Uighur population by Hardy-Weinberg equilibrium test

We compared the distribution difference of APOA5 rs662799, LPL rs320, LPL rs328, CETP rs708277 and LDLR rs688 gene loci in IS and control Han and Uighur population of Xinjiang. Then we calculated the difference in overall case and control group by Hardy-Weinberg equilibrium test. Data were shown in Tables 3 and 4. The frequencies of LDLR rs688 mutation were dramatic increased in IS patients than the controls (*P* = 0.003). This result strongly supports the association LDLR mutation with IS. However, there was no difference that the frequencies of ApoA5 rs662799, LPL rs320, LPL rs328, CETP rs708272 in Hardy-Weinberg equilibrium test.

**Table 3:** Hardy-Weinberg equilibrium test of Overall sample case group

SNP	A1	A2	AN	EN	χ2	*P*
Rs662799	G	A	30/153/225	28/157/223	0.263	0.877
Rs320	G	T	12/121/275	13/119/276	0.114	0.945
Rs328	G	C	1/58/349	2/56/350	0.574	0.768
Rs708272	A	G	81/196/131	79/201/129	0.204	0.903
Rs688	T	C	40/130/238	27/156/225	11.344	0.003

*SNP* single nucleotide polymorphism, *AN* Actual number, *EN* Expectable number

**Table 4:** Hardy-Weinberg equilibrium test of Overall sample CONTROL group

SNP	A1	A2	AN	EN	χ2	*P*
Rs662799	G	A	25/110/212	18/123/205	4.320	0.115
Rs320	G	T	13/99/235	11/102/233	0.465	0.793
Rs328	G	C	1/58/349	2/56/350	0.574	0.768
Rs708272	A	G	82/160/105	76/173/99	1.817	0.403
Rs688	T	C	14/129/204	18/121/208	1.495	0.474

*SNP* single nucleotide polymorphism, *AN* Actual number, *EN* Expectable number

### The distribution difference of ApoA5 rs662799, LPL rs320, rs328, CETP rs708277, LDLR rs688 mutation with IS and control according to their dominant, recessive and codominant genetic style by pearson χ2 and CHISP test

The next, we compared the difference of ApoA5, LPL, CETP and LDLR SNP mutation between Han and Uighur IS patients and control by pearson χ2 test. The result were shown in Tables 5 and 6. By contrast, either Uighur (Table 5) or Han population (data not shown) was highly significant when compared to respective controls in recessive genetic model. However, associations were not significant in dominant genetic model. Interest, this result also is consistent by CHISP test (Tables 6 and 7). Further, no significant association was found in dominant and codominant genetic model in stroke cases (data not shown).

**Table 5:** The distribution of different genotype of recessive between two Uighur groups

SNP	Recessive	Case	Control	χ2	*P*
Rs662799	GG:(GA + AA)	30/378 (7.4/92.6)	25/322(7.2/92.8)	0.006	0.938
Rs320	GG:(GT + TT)	12/396 (2.9/97.1)	13/334(3.7/96.3)	0.380	0.538
Rs328	GG:(GC + CC)	1/407(0.2/99.8)	3/344(0.9/99.1)	NA	NA
Rs708272	AA:(GA + GG)	81/327 (19.9/80.1)	82/265(23.6/76.4)	1.581	0.209
Rs688	TT:(CT + CC)	40/368 (9.8/90.2)	14/333(4.0/90.6)	9.399	0.002

*SNP* single nucleotide polymorphism, *N/A* no answer

**Table 6:** The distribution of different genotype of recessive between two Han groups

SNP	Recessive	Case	Control	CHISP	*P*
Rs662799	GG:(GA + AA)	21/229 (8.4/91.6)	19/179(9.6/90.4)	0.194	0.659
Rs320	GG:(GT + TT)	10/240 (4.0/96.0)	8/190(4.0/96.0)	0.000	0.983
Rs328	GG:(GC + CC)	1/249(0.4/99.6)	3/195(1.5/98.5)	NA	NA
Rs708272	AA:(GA + GG)	49/201 (19.6/80.4)	49/149(24.7/75.3)	1.713	0.191
Rs688	TT:(CT + CC)	16/234 (6.4/93.6)	4/194(2.0/98.0)	4.970	0.036

*SNP* single nucleotide polymorphism, *N/A* no answer

**Table 7:** The distribution of different genotype of recessive between two Uighur groups

SNP	Recessive	Case	Control	CHISP	*P*
Rs662799	GG:(GA + AA)	9/149 (5.7/94.3)	6/143(4.0/96.0)	0.460	0.718
Rs320	GG:(GT + TT)	2/156 (1.3/98.7)	5/144(3.4/96.6)	1.5030.220	
Rs328	GG:(GC + CC)	0/158 (0.0/100.0)	0/149(0.0/100.0)	NA	NA
Rs708272	AA:(GA + GG)	32/126 (20.3/79.7)	33/116(22.1/77.9)	0.165	0.685
Rs688	TT:(CT + CC)	24/134 (15.2/84.8)	10/139(6.7/93.3)	5.597	0.018

*SNP* single nucleotide polymorphism, *N/A* no answer

### Association of ApoA5 rs662799, LPL rs320, rs328, CETP rs708277, LDLR rs688 mutation with IS in Han and Uighur population by binary logistic regression analysis

We further compared the difference of IS patients and control by binary logistic regression analysis. It was observed significantly associated with rs688 both addictive model (TT/CC, adjusted OR = 1.47, 95% CI = 1.04–2.07, *P* < 0.01) and recessive model (TT/CT + CC, adjusted OR = 2.66, 95% CI = 1.37–5.14, *p* = 0.004) (Tables 8 and 9). In Han population, ischemic stroke was observed significantly associated with rs688 both in addictive model (TT/CC, adjusted OR = 3.27, 95% CI = 1.06–10.05, *P* < 0.05). In Uighur population, no significant association was found between gene polymorphisms and the risk of ischemic stroke (data not shown). Combined analysis of multiple gene and loci, interaction effects of LDLR rs688 C/T, ApoA5 rs662799 A/G and CETP rs708272 C/T denoted a significant influence on IS susceptibility (*p* < 0.05).

**Table 8:** Additive model analysis of different genes with stroke

SNP	*P*	OR (95% CI)
Rs662799	0.205	Ref
Rs662799 GA/AA	0.139	1.28 (0.92–1.79)
Rs662799 GG/AA	0.112	1.76 (0.88–3.54)
Rs320	0.819	Ref
Rs320 GT/TT	0.891	1.02(0.74–1.42)
Rs320 GG/TT	0.551	0.77(0.33–1.80)
Rs328	0.514	Ref
Rs328 GC/CC	0.744	0.93 (0.61–1.42)
Rs328 GG/CC	0.264	0.27(0.03–2.67)
Rs708272	0.413	Ref
Rs708272 GA/GG	0.695	0.93 (0.66–1.32)
Rs708272 AA/GG	0.195	0.76 (0.50–1.15)
Rs688	0.013	Ref
Rs688 TC/CC	0.611	0.92(0.67–1.27)
Rs688 TT/CC	0.006	2.57 (1.31–5.03)

*SNP* single nucleotide polymorphism, *OR* Odds ratio, *CI* confidence interval

**Table 9:** Recessive model analysis of different genes with stroke

SNP		*P*	OR (95% CI)
Rs662799	GG/(GA + AA)	0.745	1.10(0.62–1.96)
Rs320	GG/(GT + TT)	0.537	0.77(0.33–1.78)
Rs328	GG(GC + CC)	0.268	0.28(0.03–2.69)
Rs708272	AA/(GA + GG)	0.204	0.79(0.55–1-13)
Rs688	TT/(CT + CC)	0.004	2.66(1.37–5.14)

*SNP* single nucleotide polymorphism, *OR* Odds ratio, *CI* confidence interval

### Association of LPL rs328 and CETP rs708277 with serum lipid protein levels and clinical profile in total population

To explore the association of lipid metabolism gene SNPs with serum lipid protein levels and clinical profile, we detected major serum lipid levels, glucose, height, weight, BMI and blood Pressure with LPL rs328 and CETP.rs70877 in total population. Data were shown in Tables 10 and 11. We found that there are significant lower TG in rs328 GG than in rs328 CC and GC population (Table 10). Low SBP also was measured in CETP.rs70877 GA and AA (Table 11).

**Table 10:** Association comparison of rs328 genotype with stroke in study population

Rs328	CC		GC		GG		*P*
	*n*	Mean ± SD	*n*	Mean ± SD	*n*	Mean ± SD	
Age	640	61.4 ± 12.61	111	64.4 ± 9.3	4	61.8 ± 12.9	0.052
Height	637	1.67 ± 0.08	111	1.66.1 ± 0.08	4	1.59 ± 0.065	0.058
Weight	638	68.80 ± 10.65	110	68.28 ± 11.39	4	58.75 ± 9.11	0.162
BMI	637	24.67 ± 2.89	110	24.78 ± 3.00	4	23.06 ± 92.30	0.499
SBP	640	138.49 ± 23.29	111	137.05 ± 24.52	4	122.50 ± 16.58	0.340
DBP	640	84.52 ± 42.64	111	81.50 ± 13.84	4	72.50 ± 6.46	0.642
Glucose	640	6.25 ± 3.13	111	6.20 ± 2.99	4	7.48 ± 5.45	0.725
TG	640	2.02 ± 1.24	111	1.70 ± 0.99	4	1.48 ± 0.30	0.026
TC	640	4.34 ± 1.15	111	4.33 ± 0.85	4	4.70 ± 0.6 1	0.806
HDL	640	1.19 ± 0.66	111	1.24 ± 0.84	4	1.32 ± 0.49	0.748
LDL	639	2.66 ± 0.86	111	2.63 ± 0.77	4	2.54 ± 0.32	0.926
APOA1	640	1.24 ± 0.32	111	1.23 ± 0.22	4	1.07 ± 0.23	0.514
APOB	640	0.90 ± 0.75	111	0.85 ± 0.33	4	0.99 ± 0.18	0.714
HCY	640	9.98 ± 7.29	111	8.69 ± 4.87	4	5.50 ± 2.54	0.094
TC/HDL-C	640	4.32 ± 2.10	111	4.08 ± 1.34	4	3.78 ± 0.798	0.452
LDL-C/HDL-C	639	2.68 ± 2.08	111	2.51 ± 1.04	4	2.15 ± 0.78	0.603
ApoB/ApoA1	640	0.77 ± 0.80	111	0.71 ± 0.34	4	0.97 ± 0.35	0.677

*BMI* Body Mass Index, *SBP* Systolic blood pressure, *DBP* diastolic blood pressure, *TC* Total Cholesterol, *TG* Triglycerides, *LDL-C* low Density Lipoprotein-C, *HDL-C* High Density Lipoprotein-C, *ApoA1* Apolipoprotein–A1, *ApoB* Apolipoprotein B

**Table 11:** Association comparison of rs708272 genotype with stroke in study population

Rs708272	GG		GA		AA		*P*
	*n*	Mean ± SD	*n*	Mean ± SD	*n*	Mean ± SD	
Age	236	61.0 ± 11.67	356	62.4 ± 11.7	163	62.0 ± 11.9	0.353
Height	236	1.67 ± 0.08	354	1.66.1 ± 0.08	162	1.67 ± 0.07	0.394
Weight	235	68.61 ± 10.51	355	68.59 ± 10.70	162	68.94 ± 11.33	0.937
BMI	235	24.49 ± 2.273	353	24.76 ± 2.86	163	24.78 ± 3.23	0.467
SBP	236	141.33 ± 23.96	356	136.78 ± 24.35	163	136.72 ± 20.18	0.046
DBP	236	88.74 ± 67.74	356	81.69 ± 13.74	163	85.26 ± 13.96	0.086
Glucose	236	6.06 ± 2.51	356	6.25 ± 2.87	163	6.51 ± 4.24	0.371
TG	236	2.01 ± 1.27	356	1.95 ± 1.18	163	1.95 ± 1.20	0.803
TC	236	4.34 ± 0.97	356	4.34 ± 1.18	163	4.35 ± 1.14	0.997
HDL	236	1.22 ± 0.739	356	1.19 ± 0.65	163	1.17 ± 0.58	0.738
LDL	236	2.64 ± 0.79	356	2.64 ± 0.82	163	2.70 ± 0.968	0.718
APOA1	236	1.24 ± 0.23	356	1.22 ± 0.23	163	1.27 ± 0.50	0.124
APOB	236	0.85 ± 0.28	356	0.92 ± 0.81	163	0.89 ± 0.86	0.507
HCY	236	9.81 ± 7.176	356	9.73 ± 6.93	163	9.78 ± 6.91	0.991
TC/HDL-C	236	4.21 ± 1.60	356	4.33 ± 2.40	163	4.25 ± 1.53	0.757
LDL-C/HDL-C	236	2.59 ± 1.12	356	2.71 ± 2.59	163	2.63 ± 1.18	0.730
ApoB/ApoA1	236	0.71 ± 0.28	356	0.80 ± 0.87	163	0.76 ± 0.92	0.387

*BMI* Body Mass Index, *SBP* Systolic blood pressure, *DBP* diastolic blood pressure, *TC* Total Cholesterol, *TG* Triglycerides, *LDL-C* low Density Lipoprotein-C, *HDL-C* High Density Lipoprotein-C, *ApoA1* Apolipoprotein–A1, *ApoB* Apolipoprotein B

These results indicated that different lipid protein metabolism relative gene SNPs is associated with special clinical profile.

## Discussion

The risk factors of stroke were extensively studied [10, 20, 21]. Recent data revealed that a few protein genetic polymorphisms were significantly association with the risk for stroke [22–24]. Here, we investigated that ApoA5, LPL, CETP and LDLR gene polymorphisms in IS patients from Xinjiang region. The results provided linkage of different genetic background, life style and the occurrence of ischemic stroke.

Our study showed the presence of ApoA5 rs662799, LPL rs320, LPL rs328(Ser474 Ter), CETP rs708277 and LDLR rs688 in IS patients from Xinjiang region. Surprisingly, it was observed that rs688 genetic polymorphism had significantly more difference in IS patients than in control in recessive model analysis. This phenomenon also was seen by binary logistic regression analysis of total studied population. IS patients was observed significantly associated with rs688 both addictive model (TT/CC, adjusted OR = 1.47, 95% CI = 1.04–2.07, *P* < 0.01) and recessive model (TT/CT + CC, adjusted OR = 2.66, 95% CI = 1.37–5.14, *p* < 0.01). In Han population, ischemic stroke was observed significantly associated with rs688 both in addictive model (TT/CC, adjusted OR = 3.27, 95% CI = 1.06–10.05, *P* < 0.05). However, This phenomenon weren’t observed in Uighur population. It was well known that a single gene may have different genetic polymorphism with different kind of disease [25–28]. The same gene in one kind of disease may have diverse SNPs owing to different race and place. It was widely reported that LDLR genetic polymorphisms are associated with many disease such as essential hypertension [28], coronary artery disease (CAD) [29] and high cholesterol [30]. These SNP site were C1773T, rs2228671, rs1122608 and so on. A similar study were reported that rs688 is associated with IS patients in Taiwanese population [24]. Gao et al. revealed that rs688 increase exon12 alternative splicing and affected LDL receptor function [27]. Zhu et al. showed that rs688 promote a high serum cholesterol by modulating LDLR exon12 splicing efficiency [30]. Therefore, we speculated that rs688 firstly increase serum cholesterol and then cause atherosclerotic plaques form.

ApoA5 had similar phenomenon like LDLR. It was reported that ApoA5 T-1131C, T1259C, and IVS3 + G476A are associated with IS, DM and CAD [11, 25]. These SNPs result in high triglycerides. It also was widely observed that some gene SNPs are closely associated with special clinical profile in Xinjiang. Our study indicated that SNPs of lipid metabolism relative genes is associated with response of drug [18] Zhang et al. reported that there are significant difference in β3-adrenergic receptors (ADRβ3) gene polymorphisms rs6986132 of Han and Uighur populations in Xinjiang. They also observed the ADRβ3 rs2298423 G allele carriers increase risk for TC and LDL-C level in the Uighur populations of Xinjiang [31]. Abulizi et al. found that ApoA5 gene c553G-T polymorphism is associated with high TG levels in Han and Uighur population [32]. Our present data showed that LPL rs328 CC and GC allele people have high TG than rs328 GG allele population. People of CETP rs708277 GG allele had high SBP than it in rs708277 GA or AA allele people.

## Conclusion

Our results indicated that SNPs of lipid metabolism relative gene is closely associated with different population of Xinjiang. In Han population, ischemic stroke was significantly associated with LPLR rs688 polymorphisms. However, this phenomenon didn’t detect in Uighur population. We also found that different special SNPs allele have different serum lipid and blood pressure levels. These results demonstrated that we need take care of some specific SNPs people because they is easy to develop ischemic strokes.

## Abbreviations

ADRβ3: β3-adrenergic receptors
ApoA5: Apo lipoprotein A5
BP: Blood pressure
CETP: Cholesteryl ester transfer protein
DM: Diabetes mellitus
FPG: Fasting plasma glucose
HT: Hypertension
IFG: Impaired fasting glucose
IS: Ischemic stroke
LDL-R: Low-density lipoprotein receptor
LPL: Lipoprotein lipase
MRI: Magnetic resonance imaging
TG: Triglyceride
